# A Reinforcement-Based Learning Paradigm Increases Anatomical Learning and Retention—A Neuroeducation Study

**DOI:** 10.3389/fnhum.2018.00038

**Published:** 2018-02-06

**Authors:** Sarah J. Anderson, Kent G. Hecker, Olave E. Krigolson, Heather A. Jamniczky

**Affiliations:** ^1^Department of Community Health Sciences, Cumming School of Medicine, University of Calgary, Calgary, AB, Canada; ^2^Department of Veterinary Clinical Diagnostic Services, Faculty of Veterinary Medicine, University of Calgary, Calgary, AB, Canada; ^3^Centre for Biomedical Research, University of Victoria, Victoria, BC, Canada; ^4^Department of Cell Biology and Anatomy, Cumming School of Medicine, University of Calgary, Calgary, AB, Canada

**Keywords:** electroencephalography (EEG), event-related potential (ERP), neuroeducation, reinforcement learning, reward positivity, N250, anatomy education

## Abstract

In anatomy education, a key hurdle to engaging in higher-level discussion in the classroom is recognizing and understanding the extensive terminology used to identify and describe anatomical structures. Given the time-limited classroom environment, seeking methods to impart this foundational knowledge to students in an efficient manner is essential. Just-in-Time Teaching (JiTT) methods incorporate pre-class exercises (typically online) meant to establish foundational knowledge in novice learners so subsequent instructor-led sessions can focus on deeper, more complex concepts. Determining how best do we design and assess pre-class exercises requires a detailed examination of learning and retention in an applied educational context. Here we used electroencephalography (EEG) as a quantitative dependent variable to track learning and examine the efficacy of JiTT activities to teach anatomy. Specifically, we examined changes in the amplitude of the N250 and reward positivity event-related brain potential (ERP) components alongside behavioral performance as novice students participated in a series of computerized reinforcement-based learning modules to teach neuroanatomical structures. We found that as students learned to identify anatomical structures, the amplitude of the N250 increased and reward positivity amplitude decreased in response to positive feedback. Both on a retention and transfer exercise when learners successfully remembered and translated their knowledge to novel images, the amplitude of the reward positivity remained decreased compared to early learning. Our findings suggest ERPs can be used as a tool to track learning, retention, and transfer of knowledge and that employing the reinforcement learning paradigm is an effective educational approach for developing anatomical expertise.

## Introduction

In anatomy education, instructors utilize reinforcement learning principles informally in lecture and lab settings to build foundational knowledge. Reinforcement learning is an adaptive modification of behavior where information gathered from a learner’s previous experience, in this case, instructor feedback, is used to modify subsequent choices to maximize future performance (Sutton and Barto, 1998). This time-consuming process dominates limited student-instructor interactions since anatomy curricula requires extensive terminology to identify and describe anatomical structures. More appropriately, interactions should pursue understanding of function, inter-relation, and clinical implications of anatomy. However, acquiring foundational knowledge is a key hurdle to overcome to participate in deeper learning experiences (Pandey and Zimitat, 2007).

Just-in-Time Teaching (JiTT) methods incorporate independent pre-class exercises (typically online) to establish foundational knowledge followed by instructor-led teaching that leverages this foundation to focus on more complex concepts (Marrs and Novak, 2004). JiTT methodology, specifically pre-class exercises, should focus on incorporation and retention of knowledge into long-term memory (Custers, 2010; Mayer, 2010). The practical gap in anatomy education is how best do we design and assess pre-class exercises to foster learning and retention?

Our research operationalizes use of reinforcement learning within the context of pre-class exercises to build foundational anatomic knowledge. While using behavioral methods alone provides a basic understanding of learning processes (Anderson et al., 2016), directly measuring learning using neuroimaging can provide insight at a more detailed level (Ansari et al., 2012). JiTT pre-class modules offer a unique applied educational setting to explore measurement of neural correlates of learning.

Studies using event-related potential (ERP) methodology have quantified neurological changes in reinforcement-based learning paradigms. For example, ERP component amplitudes changed as participants learned to perform a feedback dependent perceptual categorization task (Krigolson et al., 2009). Supporting this, the N250—an ERP component associated with visual object recognition increases in amplitude during acquisition of visual perceptual expertise (Scott et al., 2006, 2008; Tanaka et al., 2006). During reinforcement learning tasks, the brain predicts reward values associated with each potential choice action. Through a feedback dependent trial and error process, the predicted value of an action comes to approximate the true value of the action, thus a learner’s chance of successfully choosing the appropriate actions increases (Sutton and Barto, 1998). The reward positivity is an ERP component associated with evaluation of performance feedback (Holroyd et al., 2008; Krigolson et al., 2009; Proudfit, 2014). As a subject gains perceptual expertise (marked by an increase in N250 amplitude) their ability to internally evaluate their responses increases and reliance on external feedback decreases (marked by a decrease in reward positivity amplitude) (Krigolson et al., 2009). Further, it was found that reward positivity amplitude scaled like a prediction error–suggesting reward positivity amplitude changes are linked to differences between expected and actual rewards; as a subject *learns*, there will be a diminished amplitude in response to the positive feedback (Krigolson et al., 2014). In controlled settings (of N250 and reward positivity), examination of retention has been limited to a scale of hours to several days (Scott et al., 2008; Arbel et al., 2013). Designing studies that permit examination of neural signals during initial learning, retention and transfer exercises is essential to determining whether neural correlates can be used as a tool to track learning and memory in applied educational settings.

The objective of this study was to examine amplitude changes of the reward positivity and N250 as quantitative dependent variables to evaluate reinforcement-based JiTT pre-class exercises. We predicted that as participants learned to identify and localize neuroanatomical structures: (a) N250 amplitude would increase and remain elevated during retention and transfer exercises; (b) reward positivity amplitude would decrease as ability to internally evaluate the correctness of responses enhances; (c) changes in reward positivity would be greatest in the first learning exercise when information is novel as opposed to retention exercises; and (d) diminished reward positivity amplitude will be maintained as participants translate knowledge to a new context.

## Materials and Methods

### Participants

Twenty-three participants (11 females, 12 males; mean age = 20.74 years (SD = 2.30)) were recruited from anatomy classes at the University of Calgary, Calgary, AB, Canada. The experiment was run in two similar cohorts of students in anatomy courses (academic programs included Bachelor of Health Sciences and Biomedical Engineering). Participation was voluntary and written informed consent was obtained in accordance with the Declaration of Helsinki. Participants had minimal neuroanatomical knowledge related to cranial nerve identification, as this was not part of curriculum taught thus far in the anatomy classes. Prior to the experimental task, this was confirmed using an identification test on information to be learned in the task (no participant performance exceeded 75% accuracy, thus no exclusions were made). All participants had normal, or corrected-to-normal vision, and no known neurological impairments. This study was approved by the Conjoint Health Research Ethics Board at the University of Calgary (Ethics ID: REB14-088).

### Experimental Design

**Figure 1:**
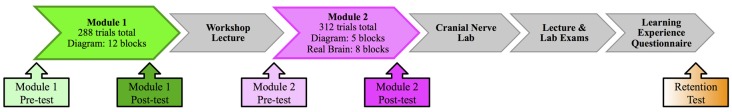
Sequence of learning events.

Participation in this experiment involved completion of a multi-session learning module to teach human cranial nerve anatomy. The overall module consisted of two computer-based sessions that bounded an in-class workshop lecture followed by a laboratory session (see Figure 1 for an outline of the sequence of learning events). Electroencephalography (EEG) data was acquired during computer-based modules as participants completed reinforcement-based learning tasks (outlined below) meant to teach identification and localization of the cranial nerves. Participants were administered an identification test prior to and following this task to assess knowledge. The in-class workshop consisted of a small group activity where students were assigned sets of cranial nerve lesion cases, where they had to identify the nerve involved and surrounding considerations associated with the particular injury. This was followed by a whole group presentation and discussion of the cases. In the laboratory session participants examined cadaveric brains and skulls to review information and gain appreciation for the close spatial relationships of anatomical structures. Finally, a long-term retention test was administered to the first cohort (*n* = 11) of students at approximately 20 weeks following the learning exercises.

### Reinforcement-Based Learning Module

During the computer-based learning module, participants were seated comfortably in front of a 17″ ASUS laptop computer and provided a standard USB gamepad to respond. Questions were presented during the task on the computer screen using a customized MATLAB (Release 2015a, MathWorks, Natick, MA, USA) script in conjunction with Psychophysics Toolbox extensions (Brainard, 1997; Pelli, 1997). Framework for this experimental task was adapted from Krigolson et al. (2009), and a detailed description of this task was previously described in Anderson et al. (2016).

Across a series of trials (questions), participants were trained to identify and localize 12 cranial nerves through a trial and error shaping process based on positive and negative feedback provided. For each cranial nerve three incorrect labels were purposefully selected from the other 11 possible nerves such that all 12 nerve labels were equally used and served as proximal distractors for the correct label. On each question the correct label was shown 50% of the time while one of the three incorrect labels was shown the other 50% of the time. A trial consisted of the following components: a fixation cross (500 ms); an image of a brain with an arrow indicating a cranial nerve (1500 ms); a label for the cranial nerve (50% chance of being correct), and a response by participants indicating “correct” or “incorrect” using the gamepad (maximum time allowed 2000 ms). Accuracy feedback was then provided to the participant in the form of an “✗” for incorrect trials or “✓” for correct trials. Based on this feedback participants were expected to attempt to modify future responses. Trials (approximately 5 s) were grouped into blocks of 24 trials (approximately 2 min), participants were provided a rest period following each block, and could advance to the next block when ready. We collected accuracy and response time information for each trial to construct learning curves. On trials where participants were too slow to respond to questions (0.72% of total trials), we assumed that the participant would have been incorrect, and the maximum time allowed for a response (2000 ms) was used as a response time for analysis purposes.

**Figure 2:**
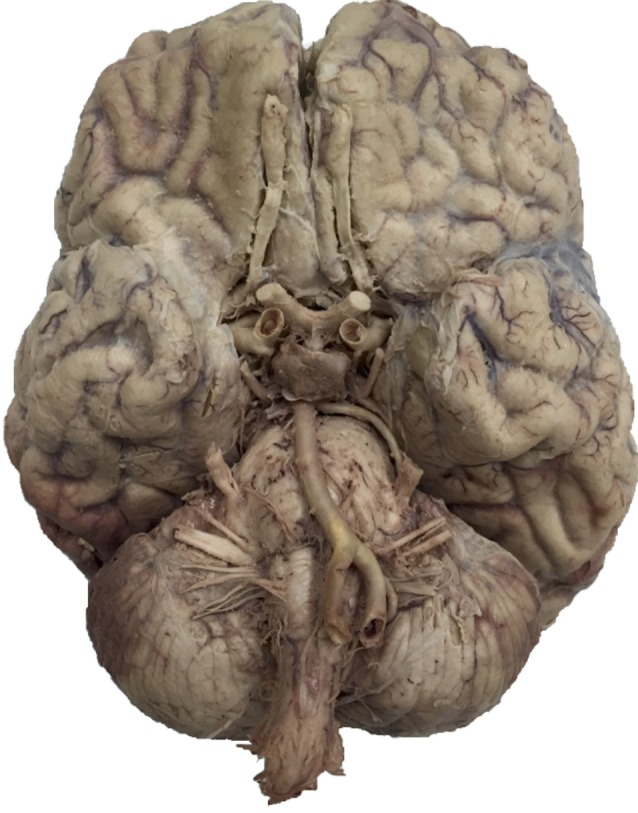
Example of photo brain image (from the University of Calgary Anatomical Specimens Collection).

The first computer-based session employed a diagrammatic representation of the brain (image modified from Thieme Teaching Assistant, Baker et al., 2015) and the task consisted of 12 blocks (288 total trials). The second session began with the diagrammatic representation for five blocks, then switched to an image of a real brain (from the University of Calgary Anatomical Specimens Collection) for the remaining eight blocks (312 total trials). See Figure 2 for an example of the real brain image used. Each of the computer-based learning tasks were completed over approximately 30 min.

### Electroencephalographic Data Acquisition and Analysis

We recorded EEG data during the computer-based learning modules from 16 electrode locations (FP1, FP2, AFz, FC5, FC6, FCz, C3, C4, TP9, P3, Pz, P4, TP10, POz, O1, O2, plus ground and reference) with an actiCAP Xpress acquisition system arranged in a standard 10–20 layout (Brain Products, GmbH, Munich, Germany) and Brain Vision Recorder Software (Version 1.20, Brain Products, GMbH, Munich, Germany). Electrode impedances were kept below 20 kΩ. EEG data was sampled at a rate of 500 Hz and amplified (V-Amp, Brain Products, GmbH, Munich, Germany: 0–500 Hz bandwidth, 24-bit A/D conversion).

We conducted offline EEG data analysis using Brain Vision Analyzer 2 software (Version 2.1, Brain Products, GmbH, Munich, Germany). EEG analysis steps were performed as follows (for more details, see http://www.neuroeconlab.com/data-analysis.html). First, any overly noisy electrodes were removed. Second, data were down-sampled to a rate of 250 Hz to make the file sizes smaller for enhanced computer analysis processing speed. Third, the data were re-referenced from a common reference to linked TP9 and TP10 electrodes. Next, the data were filtered using a phase shift-free Butterworth filter with a 0.1–30 Hz passband and a 60 Hz notch filter.

Next, epochs for each ERP component of interest were created. For the N250, the event of interest was the appearance of the brain image in early trials (first 48 trials) vs. late trials (last 48 trials). For reward positivity, events were linked to feedback onset and split by valence (correct or incorrect responses) as well as early correct (first 50 correct feedback trials) vs. late correct trials (last 50 correct feedback trials).

Data was segmented into 3000 ms units surrounding the event of interest (1000 ms before and 2000 ms after) in order to performed independent component analysis to correct for ocular artifacts. Data was then re-segmented into 800 ms epochs surrounding events (200 ms before and 600 ms after) and baseline corrected using the mean voltage calculated from the 200 ms preceding the event. We removed artifacts from the data if voltage on any channel exceeded 10 μV/ms gradient and 150 μV absolute difference criteria.

ERP waveforms were created by averaging EEG epochs across all participants for each experimental condition outlined above. We defined the N250 ERP component as the mean voltage from 230 ms to 330 ms following presentation of the stimulus at electrode site O1. As in previous research, the latency window was selected based on visual inspection of the grand average waveform and electrode site was reported for where the N250 amplitude was maximal (Scott et al., 2006, 2008; Tanaka et al., 2006; Krigolson et al., 2009). Difference waveforms were constructed by subtracting ERPs on early trials from late trials to examine development of perceptual expertise during the learning modules.

We measured reward positivity as voltage potential changes measured maximally at the FCz electrode site (overlying the medial frontal cortex) in line with previous research (Holroyd and Coles, 2002; Holroyd and Krigolson, 2007; Krigolson et al., 2009). Reward positivity was defined as the mean voltage of averaged waveforms from 264 ms to 304 ms post feedback at electrode site FCz. To compare response to positive vs. negative feedback, we constructed a difference waveform by subtracting the ERP on incorrect trials from the ERP on correct trials for each participant (Proudfit, 2014). Previous research suggests that reward positivity is reduced with learning (Krigolson et al., 2009, 2014), thus we also examined changes in reward positivity in response to positive feedback across the entire experiment. We generated six grand averaged ERP waveforms (module one (diagram-based learning) early and late; module two (diagram-based learning) early and late; and module two (real image-based learning) early and late).

### Statistical Analysis

Statistical analysis was performed using SPSS Statistics (Version 24). Statistical comparisons were made using paired sample *t*-tests to examine behavioral (pre and post knowledge test) and ERP component changes (N250 and reward positivity (correct vs. incorrect feedback) under each condition assuming an alpha level of 0.05 for statistical significance. Repeated measures analysis of variance (RM-ANOVA) was used to compare behavioral changes (accuracy across blocks) and reward positivity changes across the entire experiment. Bonferonni *post hoc* analysis was completed to identify specific differences. An additional Greenhouse-Geisser correction was applied to adjust the degrees of freedom as the assumption of sphericity was violated in the behavioral accuracy analysis. Equal variance across the blocks in accuracy performance did not occur due to the ceiling effects innate to learning data. Effect sizes were determined by calculating Cohen’s (1988); *d* (*t*-tests) and partial eta squared (RM-ANOVA) to demonstrate magnitude of effect.

## Results

### Behavioral Analysis

#### Computer-Based Learning Module 1

**Figure 3:**
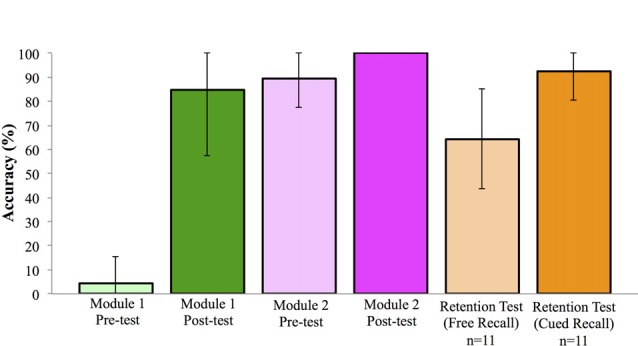
Performance on knowledge test across learning events (±SD). Note: all participants achieved 100% in the Module 2 Post-test.

Prior to participating in any learning activities, participants completed a test, employing a diagrammatic brain representation, to assess prior knowledge of location and identification of cranial nerves. Module 1 Pre-test score was 4.17%, 95% CI [−0.66, 9.00]. Following completion of the session, there was a significant increase in performance on the Module 1 Post-test score, *t*_(22)_ = 14.14, *p* < 0.001, *d* = 3.86, paired *t*-test, mean accuracy of 84.78%, 95% CI [72.95, 96.62]. Knowledge test performance across the experiment is shown in Figure 3.

**Figure 4:**
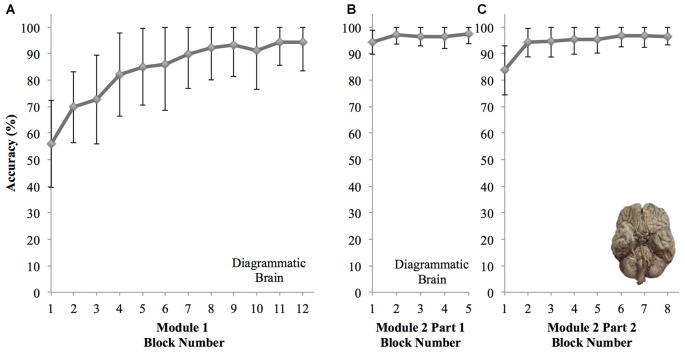
Graphs showing changes in mean accuracy performance for each block of the reinforcement based learning computer modules for all participants (±SD). **(A)** Module 1—diagrammatic brain image used for 12 blocks showing novice learning. **(B)** Module 2 Part 1—diagrammatic brain image used for five blocks showing knowledge retention. **(C)** Module 2 Part 2—photo image of brain used for eight blocks showing transfer of knowledge.

We observed that participant learning curves indicate the proportion of correct responses increases over 12 blocks using the diagram image. The averaged learning curve for all participants is shown in Figure 4A. There was a significant effect of block number on cranial nerve identification performance, *F*_(4.94,108.71)_ = 45.93, *p* < 0.001, RM-ANOVA, partial eta squared = 0.68. Mean accuracy in the first block was 55.98%, 95% CI [48.90, 63.06]. Mean accuracy consistently improves across each block to 94.20%, 95% CI [89.54, 98.87] in the final block, and from block 6 (144 trials) onward, no significant changes in performance were observed (*p*’s > 0.05).

#### Computer-Based Learning Module 2

Following the in-class workshop, participants returned to complete the second computer-based learning module. Participation in the second module was 6.87 days (SD = 3.38) following the first module. The second session began with a repetition of the identification test, using a diagrammatic brain representation to assess retention of prior information. Mean score on this test was 89.67%, 95% CI [84.33, 95.02]. Following the session, a new identification test was administered, using an image of a real brain; here all students achieved a score of 100.00% (Figure 3).

Part one of the second learning module consisted of five blocks of trials using the diagrammatic brain representation (Figure 4B). Here, block number had no significant effect on cranial nerve identification performance, *F*_(3.07,67.61)_ = 2.58, *p* = 0.06, RM-ANOVA, partial eta squared = 0.11. Performance remained consistently high ranging from 94.38%, 95% CI [92.38, 96.39], on the first block to 97.46%, 95% CI [95.86, 99.07] on the last block. The second part of the module consisted of eight blocks of trials using an image of a real brain (Figure 4C). Block number had a significant effect on performance, *F*_(3.86,84.89)_ = 18.62, *p* < 0.001, RM-ANOVA, partial eta squared = 0.46. Specifically, performance was significantly lower on the first block (mean accuracy 83.70%; 95% CI [79.67, 87.72], *p*’s < 0.001). However performance on the second and remaining blocks did not differ significantly (*p*’s > 0.05), and the mean accuracy in the final block was 96.56%, 95% CI [95.16, 97.96].

#### Comparison of Performance Across Experiment

From the post-test of the first module to the retention test in the second module, mean performance improved by 4.89%; however this increase was not statistically significant, *t*_(22)_ = 1.05, *p* = 0.31, *d* = 0.23, paired *t*-test. When a second retention test was administered to the first cohort (*n* = 11) at approximately 20 weeks following the learning module, some knowledge was retained. Here, on a free recall identification and localization test participants achieved a mean score of 64.39%, 95% CI [50.49, 78.30], while on a cued recall test (where a list of potential cranial nerves was provided) mean scores significantly improved to 92.42%, 95% CI [84.43, 100.42]; *t*_(10)_ = 6.60, *p* < 0.001, *d* = 1.66, paired *t*-test (Figure 3).

There is no significant lapse in accuracy performance from the end of the first to the second learning sessions (*p* = 1.00). Interestingly, in part two of the second session when the representation employed transitioned to a real image of a brain, a drop in performance was only observed in the first block (*p* < 0.001), however performance in subsequent blocks of part two did not differ significantly from the earlier five blocks of the session that used the diagrammatic brain representation (*p*’s > 0.05).

#### Response Time

Block number had a significant effect on response time in all modules (Module 1: *F*_(11,6061)_ = 157.83, Module 2 (Part 1): *F*_(7,3857)_ = 74.08; Module 2 (Part 2): *F*_(4,2204)_ = 15.84; all *p*’s < 0.001, RM-ANOVA, partial eta squared = 0.03–0.22). Generally response time significantly decreased following the first block of each module (Module 1, Module 2 Part 1 and Module 2 Part 2) and response times followed a decreasing trend across each module.

### Electroencephalographic Data

The N250 and reward positivity ERP components were independently analyzed for each of the experimental conditions during the computer-based learning sessions. Due to either excessively noisy electrodes of interest and/or the excessive presence of artifacts 4 of the 23 participants were removed (>79% of trials discarded). The remaining data (19 participants) were retained for subsequent analysis (<12% artifacts).

#### Object Recognition: The N250

**Figure 5:**
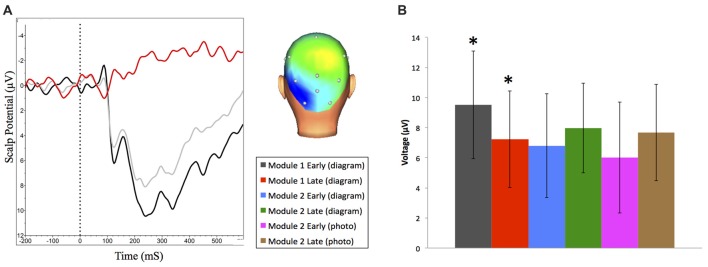
**(A)** Grand averaged N250 event-related brain potential (ERP) waveforms at O1 and scalp distribution (between 230 ms and 330 ms) for early (dark) and late (light) trials during Module 1. ERP difference waveform (red) shows late minus early trials. Negative is plotted up. **(B)** Mean N250 amplitude (between 230 ms and 330 ms) at O1 across all learning events (±95% CI). *N250 amplitude significantly decreases during Module 1.

Examination of the ERPs averaged to the onset of presentation of the brain images revealed a left unilateral posterior N250 (maximal at channel O1). The N250 at O1 significantly increased in amplitude from the start to the finish of the first learning module, *t*_(18)_ = 2.45, *p* = 0.03, *d* = 0.32, paired *t*-test (Figure 5). In the second learning module there was no significant change in N250 amplitude from early to late trials in either the diagram-based learning (*t*_(18)_ = 1.25, *p* = 0.23, *d* = 0.18, paired *t*-test) or real image-based learning (*t*_(17)_ = 1.59, *p* = 0.13, *d* = 0.24, paired *t*-test) portions of the module.

#### Feedback Processing: The Reward Positivity

**Figure 6:**
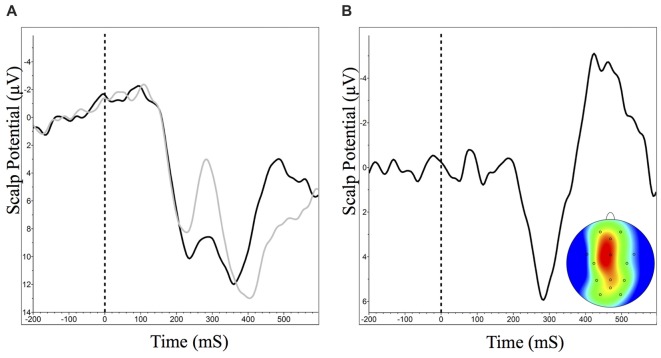
Grand averaged reward positivity ERP waveforms at FCz and scalp distribution for accuracy feedback in Module 1. Reward positivity is maximal between 264 ms and 304 ms following feedback presentation. Negative is plotted up. **(A)** ERP waveform at FCz in response to correct (dark) and incorrect (light) feedback. **(B)** ERP difference waveform and peak scalp distribution for correct minus incorrect accuracy feedback.

Difference wave analysis on responses to correct vs. incorrect feedback was only possible for the first learning session, since beyond this session participant performance was too high to have enough error trials to generate ERP waveforms for response to receiving incorrect feedback. Analysis revealed an ERP component consistent with previously measured reward positivity, and was maximal at the FCz channel with a peak latency of 284 ms (Figure 6), *t*_(17)_ = 4.50, *p* < 0.001, *d* = 1.05, paired *t*-test.

**Figure 7:**
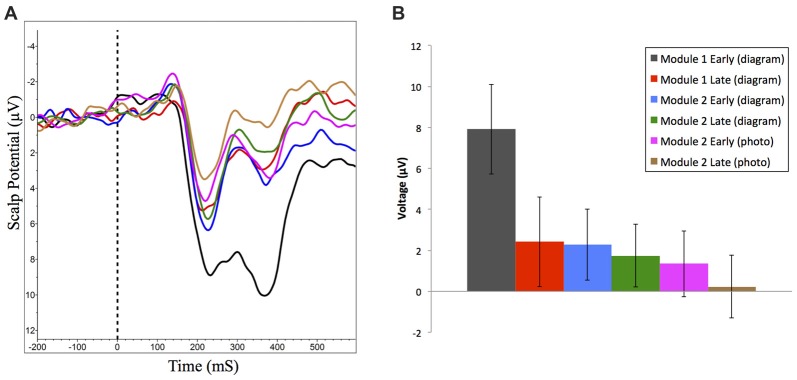
**(A)** Grand averaged reward positivity ERP waveforms at FCz in response to correct accuracy feedback across all learning events. Negative is plotted up. **(B)** Mean reward positivity amplitudes (between 264 ms and 304 ms) at FCz (±95% CI). Reward positivity amplitude is significantly greater than all other time points in early learning of Module 1.

We also examined changes in amplitude of reward positivity in response to positive feedback across the experiment. To generate these ERP waveforms, trials where participants received feedback indicating they were correct were extracted from the first and last 50 trials of each experimental condition. The following ERP waveforms were generated: module one (diagram-based learning) early and late; module two (diagram-based learning) early and late; and module two (real image-based learning) early and late (Figure 7A). Amplitudes of each waveform were compared from 264 ms to 304 ms at FCz. Experimental condition had a significant effect on amplitude of response to positive feedback, *F*_(5,90)_ = 10.99, *p* < 0.001, RM-ANOVA, partial eta squared = 0.38. Specifically, the amplitude was significantly greater for the early set of trials for the first module (*p*’s between <0.001 and 0.006), but all other trial sets did not significantly differ from each other (*p*’s > 0.05; Table 1, Figure 7B).

**Table 1:** Reward positivity across learning modules in response to correct feedback (μV).

Module time point		Mean	Standard deviation	95% Confidence interval	
				Lower bound	Upper bound
Module 1 (Diagram)	Early	7.91	4.53	5.73	10.09
	Late	2.41	4.53	0.23	4.60
Module 2 Part 1 (Diagram)	Early	2.27	3.61	0.53	4.01
	Late	1.74	3.17	0.21	3.27
Module 2 Part 2 (Photo)	Early	1.34	3.35	−0.27	2.96
	Late	0.23	3.20	−1.31	1.78

## Discussion

Successful learning is, in essence, the result of changes in brain activity related to the proper storage and retrieval of information. The objective of this study was to measure these neural changes and use them as a new quantitative dependent variable from which to examine learning in an applied educational setting. Our results demonstrate that as novice participants learn to identify anatomical structures: (a) N250 amplitude (a marker of perceptual expertise) is enhanced; (b) reward positivity amplitude (a signal associated with reinforcement learning systems in response to positive feedback) decreases; (c) the diminished reward positivity amplitude to positive feedback is maintained on a knowledge retention exercise; and (d) reward positivity amplitude remains diminished as learners successfully transfer their learning to a new context. Together the behavioral and neural correlate evidence indicate that JiTT activities employing a reinforcement learning paradigm are an effective method to build retainable knowledge.

Our results show a significant elevation in N250 amplitude as participants successfully learn to identify neuroanatomical structures. The N250 component was lateralized to the left occipital region, which was also reported by Pierce et al. (2011) who suggested that activation in this region was related to accessing stored representations in visual memory structures. Interestingly, we did not detect a significant change in N170 amplitude (an ERP component associated with recognition at a basic level) over this same period. The simplest explanation for this is that while participants learned our task they were not yet experts due to an insufficient amount of practice (Ericsson, 2009).

In accordance with the behavioral data indicating a high level of knowledge retention and transfer during the second learning module, N250 did not differ from the end of the first module to the beginning of the second module or within each of the early to late trials of the retention and transfer exercises. In a task where participants learned to categorize cars, Scott et al. (2008) noted a similar persistence of the N250 1 week following training. The authors suggested that the persistence of the N250 signal is related to acquisition of long-term perceptual expertise. In our tasks, stimuli consisted of the same image with an arrow indicating 12 different regions, such that participants experience a large amount of repetition; this is also the case in the Scott et al. (2008) study where 60 car stimuli were used as part of eight training sessions prior to the retention task. In an earlier study, Scott et al. (2006) note that the second presentation of the identical stimuli results in a smaller N250 compared to the first presentation of the stimuli during subordinate level perceptual training.

We observed a significant decrease in reward positivity amplitude over the course of the first module where participants successfully learn to identify neuroanatomical structures based on feedback. Our finding is consistent with the previously described reward positivity ERP component where the ERP component is focussed over the medial frontal cortex (Krigolson et al., 2009, 2014; Proudfit, 2014). Evidence provided by Krigolson et al. (2014) suggests that changes in reward positivity measured at the scalp level reflect changes in a reinforcement learning prediction error (the difference between actual vs. expected values of a reward). This work provides further evidence that, as a subject learns, their ability to internally evaluate their own responses is enhanced, and thus their reliance upon, and neural response to, externally provided feedback will decrease (Krigolson et al., 2009, 2014).

Our results further support the use of a reinforcement learning paradigm as a robust method for developing *retainable* expertise and that reward positivity can be used as a tool to track change and retention in learning. Specifically here we extend the measurement of retention to approximately 1 week, and report both continued maintenance of reward positivity signal trends and supporting behavioral data indicating retention of knowledge.

When participants were shown a new stimulus in the second module (a photo of a real brain) and asked to identify structures, behavioral performance significantly dropped in the first block (mean accuracy 83.70%; SD = 9.31) however reward positivity signal was maintained. This indicates that despite a drop in behavioral performance, neural data indicates participants are still able to self evaluate responses prior to external feedback. Further support for this theory is that behavioral performance rebounded to previous accuracy levels by the second block (from late trials of diagram based learning in Module 2 Part 1) and reward positivity signals continue to trend downwards by completion of the module. This suggests that learning in the new context built upon knowledge from the prior context (related to the diagram) rather than beginning from a novice level. This is an important finding since it is a clear example of the potential value of neuroeducational approaches in empirically accessing learning processes that behavioral data alone cannot reveal. Measuring neural changes related to learning offers the opportunity to define neural correlates as markers linking a learner’s behavioral performance to changes in neural signals and thus the capacity to directly explore a learner’s cognitive processing without interfering with the actual processing itself (Dalgarno et al., 2010). Furthermore, changes in neural correlates can be observed before the differences in behavioral outcomes are apparent. This offers access to earlier stages of learning and provides an alternative measure for testing the efficacy of different teaching methods in a quantitative manner to inform advancements in instructional design (Dalgarno et al., 2010; Friedlander et al., 2011; Della Sala and Anderson, 2012).

Reinforcement-based approaches to studying have been shown to enhance recall of information over time compared to studying alone in recognition-based information learning tasks (Schmidt and Bjork, 1992; Roediger and Butler, 2011). Here we have formalized reinforcement-based learning as a strategy to impart and promote retention of anatomic knowledge in novice learners. By collecting neural data in addition to behavioral data we are better able to capture the nuances in learning and provide empirical evidence for success of this strategy. Leveraging reinforcement learning as an explicit JiTT activity to teach foundational knowledge in anatomy education then allows for student-instructor interactions to advance to clarification and elaboration of knowledge.

This work serves as a starting point to study the applied use of EEG as a tool to track learning in an applied context. Given the exploratory nature of this work, further investigations are required into the following areas. First, the period between the first and second module we utilized was 6.87 days (due to academic course constraints). The “retention interval”, though greater than reward positivity studies and in line with perceptual expertise studies (Scott et al., 2008), is not very long on the spectrum of what is considered successful retention in an educational setting. Typical retention intervals in educational research are 3–4 weeks. We were able to administer a behavioral identification test at 20 weeks in a sub-cohort of our participants to explore long-term retention and this did show promising behavioral results (particularly for a cued response). Future studies will include examination of neural signals at intervals more in line with educational research on retention. Second, a workshop between the two computer based modules may have affected learning and memory processes. Despite the emphasis in the workshop on clinical relevance of lesions to the cranial nerves (rather than localizing the nerves on specimens), this discussion likely promoted rehearsal of the names of the nerves and contributed to consolidation of knowledge. Third, in the first module two participants did not achieve the same accuracy levels as the rest of the group and could be considered low learners. We did not split the analysis of the two low learners from the first module out from the group performance to examine ERPs, since performance of these two learners did not differentiate from the group in the second module. To more closely examine factors contributing to the low learner status, we would need to gather enough participants to stratify learners to two groups. Finally, as with any educational intervention these findings may be context specific. Further work to explore different anatomical regions and image fidelity in other modules would add to the generalizability of these findings.

While this work is meant to be foundational in nature by exploring the feasibility of measuring neural correlates in an educational setting, it opens potential avenues of application in teaching practice. Because neural data allows earlier access to learning processes compared to behavioral data, measurement of neural correlates could be used to compare teaching methods or types of anatomical representations to best promote efficiency in learning. On an individual level, there is potential to identify learners at risk using neural data. If a learner is not demonstrating the expected changes in neural signals associated with successful learning this could indicate challenges with initial learning and be predictive of future problems with retention. Specifically, the neural information could be used to differentiate between deficiencies in visual recognition—if there is an absence of N250 amplitude changes, or challenges related to internalizing and applying feedback—if RewP amplitude remains elevated; this would allow for targeted intervention by an instructor.

In conclusion, engaging in higher-level discussion in the classroom often requires that students possess foundational knowledge from which to explore. Given the time-limited classroom environment, seeking methods to impart this knowledge to students in an efficient manner is essential to permit subsequent interactive exploration of content. This study describes an evidence-based approach to successfully instil foundational knowledge combined with a neuroscientific approach to measure and quantify results. These findings give insight into the biology of learning in an applied setting and demonstrate the utility of measuring neural correlates event related potentials (N250 and reward positivity) to reveal learning processes underlying behavioral performance.

## Author Contributions

SJA, KGH, OEK and HAJ conceived and designed the research, interpreted the data, critically reviewed the manuscript and ensured the integrity of this work. SJA collected and analyzed the data and drafted the manuscript.

## Conflict of Interest Statement

The authors declare that the research was conducted in the absence of any commercial or financial relationships that could be construed as a potential conflict of interest.

## References

[B1] Anderson S. J. Krigolson O. E. Jamniczky H. A. Hecker K. G. (2016). Learning anatomical structures: a reinforcement-based learning approach. Med. Sci. Educ. 26, 123–128. 10.1007/s40670-015-0219-2

[B2] Ansari D. De Smedt B. Grabner R. H. (2012). Neuroeducation—a critical overview of an emerging field. Neuroethics 5, 105–117. 10.1007/s12152-011-9119-3

[B3] Arbel Y. Goforth K. Donchin E. (2013). The good, the bad, or the useful? The examination of the relationship between the feedback-related negativity (FRN) and long-term learning outcomes. J. Cogn. Neurosci. 25, 1249–1260. 10.1162/jocn_a_00385 23489147

[B4] Baker E. W. Schuenke M. Schulte E. (2015). Anatomy for Dental Medicine. New York, NY: Thieme.

[B5] Brainard D. H. (1997). The psychophysics toolbox. Spat. Vis. 10, 433–436. 10.1163/156856897x00357 9176952

[B6] Cohen J. (1988). Statistical Power Analysis for the Behavioral Sciences Lawrence Earlbaum Associates. 2nd Edn. Hillsdale, NJ: Lawrence Erlbaum.

[B7] Custers E. J. (2010). Long-term retention of basic science knowledge: a review study. Adv. Health Sci. Educ. Theory Pract. 15, 109–128. 10.1007/s10459-008-9101-y 18274876

[B8] Dalgarno B. Kennedy G. Bennett S. (2010). Can functional brain imaging be used to explore interactivity and cognition in multimedia learning environments? J. Interact. Learn. Res. 21, 317–342. Available online at: https://www.learntechlib.org/p/29543/

[B9] Della Sala S. Anderson M. (2012). Neuroscience in Education: The Good, The Bad, and The Ugly. Oxford: Oxford University Press.

[B10] Ericsson K. A. (2009). Development of Professional Expertise: Toward Measurement of Expert Performance and Design of Optimal Learning Environments. New York, NY: Cambridge University Press.

[B11] Friedlander M. J. Andrews L. Armstrong E. G. Aschenbrenner C. Kass J. S. Ogden P.. (2011). What can medical education learn from the neurobiology of learning? Acad. Med. 86, 415–420. 10.1097/ACM.0b013e31820dc197 21346504

[B12] Holroyd C. B. Coles M. G. H. (2002). The neural basis. of human error processing: reinforcement learning, dopamine, and the error-related negativity. Psychol. Rev. 109, 679–709. 10.1037/0033-295X.109.4.679 12374324

[B13] Holroyd C. B. Krigolson O. E. (2007). Reward prediction error signals associated with a modified time estimation task. Psychophysiology 44, 913–917. 10.1111/j.1469-8986.2007.00561.x 17640267

[B14] Holroyd C. B. Pakzad-Vaezi K. L. Krigolson O. E. (2008). The feedback correct-related positivity: sensitivity of the event-related brain potential to unexpected positive feedback. Psychophysiology 45, 688–697. 10.1111/j.1469-8986.2008.00668.x 18513364

[B15] Krigolson O. E. Hassall C. D. Handy T. C. (2014). How we learn to make decisions: rapid propagation of reinforcement learning prediction errors in humans. J. Cogn. Neurosci. 26, 635–644. 10.1162/jocn_a_00509 24168216

[B16] Krigolson O. E. Pierce L. J. Holroyd C. B. Tanaka J. W. (2009). Learning to become an expert: reinforcement learning and the acquisition of perceptual expertise. J. Cogn. Neurosci. 21, 1833–1840. 10.1162/jocn.2009.21128 18823237

[B17] Marrs K. A. Novak G. (2004). Just-in-time teaching in biology: creating an active learner classroom using the internet. Cell Biol. Educ. 3, 49–61. 10.1187/cbe.03-11-0022 22039345 PMC3203712

[B18] Mayer R. E. (2010). Applying the science of learning to medical education. Med. Educ. 44, 543–549. 10.1111/j.1365-2923.2010.03624.x 20604850

[B19] Pandey P. Zimitat C. (2007). Medical students’ learning of anatomy: memorisation, understanding and visualisation. Med. Educ. 41, 7–14. 10.1111/j.1365-2929.2006.02643.x 17209887

[B20] Pelli D. G. (1997). The VideoToolbox software for visual psychophysics: transforming numbers into movies. Spat. Vis. 10, 437–442. 10.1163/156856897x00366 9176953

[B21] Pierce L. J. Scott L. S. Boddington S. Droucker D. Curran T. Tanaka J. W. (2011). The n250 brain potential to personally familiar and newly learned faces and objects. Front. Hum. Neurosci. 6:111. 10.3389/fnhum.2011.00111 22059071 PMC3204460

[B22] Proudfit G. H. (2014). The reward positivity: from basic research on reward to a biomarker for depression. Psychophysiology 52, 449–459. 10.1111/psyp.12370 25327938

[B23] Roediger H. L. III. Butler A. C. (2011). The critical role of retrieval practice in long-term retention. Trends Cogn. Sci. 15, 20–27. 10.1016/j.tics.2010.09.003 20951630

[B24] Schmidt R. A. Bjork R. A. (1992). New conceptualizations of practice: common principles in three paradigms suggest new concepts for training. Psychol. Sci. 3, 207–217. 10.1111/j.1467-9280.1992.tb00029.x

[B25] Scott L. S. Tanaka J. W. Sheinberg D. L. Curran T. (2006). A reevaluation of the electrophysiological correlates of expert object processing. J. Cogn. Neurosci. 18, 1453–1465. 10.1162/jocn.2006.18.9.1453 16989547

[B26] Scott L. S. Tanaka J. W. Sheinberg D. L. Curran T. (2008). The role of category learning in the acquisition and retention of perceptual expertise: a behavioral and neurophysiological study. Brain Res. 1210, 204–215. 10.1016/j.brainres.2008.02.054 18417106

[B27] Sutton R. S. Barto A. G. (1998). Reinforcement Learning: An Introduction. Cambridge, MA: MIT Press.

[B28] Tanaka J. W. Curran T. Porterfield A. L. Collins D. (2006). Activation of preexisting and acquired face representations: the N250 event-related potential as an index of face familiarity. J. Cogn. Neurosci. 18, 1488–1497. 10.1162/jocn.2006.18.9.1488 16989550

